# Massive periorbital edema following hematopoietic stem cell transplantation

**DOI:** 10.1016/j.ajoc.2022.101559

**Published:** 2022-04-30

**Authors:** Jeannette Y. Stallworth, Jonathan C. Horton

**Affiliations:** a University of California San Francisco, Department of Ophthalmology, San Francisco, CA, 94158 USA

**Keywords:** Periorbital edema, Transplant-associated thrombotic microangiopathy, Engraftment syndrome, Neuroblastoma, Autologous hematopoietic stem cell transplantation

## Abstract

**Purpose:**

To describe a case of severe, bilateral periorbital edema after hematopoietic stem cell transplantation.

**Observations:**

A three-year old girl with metastatic neuroblastoma underwent the second of two tandem autologous peripheral blood stem cell transplants, complicated by engraftment syndrome. On post-engraftment day 11, she developed acute onset of severe periorbital edema. She was soon thereafter diagnosed with transplant-associated thrombotic microangiopathy with significant volume overload requiring treatment with eculizumab and etanercept. Periorbital edema resolved after four days with concurrent treatment of her underlying condition.

**Conclusions and Importance:**

We report an ocular manifestation related to complications of hematopoietic stem cell transplantation. This highlights a non-infectious etiology of eyelid swelling in the post-transplant, immunocompromised population.

## 1 Introduction

Autologous hematopoietic stem cell transplantation has emerged as common strategy for the treatment of leukemia, lymphoma, and certain solid tumors. It allows more aggressive treatment with chemotherapy, because the bone marrow can be rescued by infusion of previously harvested progenitor cells. Prior to consolidation therapy, stem cells are collected through apheresis by administering granulocyte colony stimulating factor to induce their release from the bone marrow into the peripheral blood. In 2018, more than 22,000 hematopoietic stem cell transplants were performed in the United States.^1^

Two syndromes have been recognized as potential complications of hematopoietic stem cell transplantation: Engraftment Syndrome (ES) and Transplant-Associated Thrombotic Microangiopathy (TA-TMA). ES occurs around the time that the white cell count begins to rise, signifying successful repopulation of the bone marrow, on average seven days after stem cell transplantation.^2^ It is characterized by non-infectious fever, skin rash, capillary leak, pulmonary edema, and fluid overload. TA-TMA results from endothelial cell injury and activation of the complement system. It produces a pro-coagulant state, whereby activation of the immune system leads to microangiopathic hemolytic anemia and thrombotic thrombocytopenia. An increase in capillary permeability can result in interstitial edema and weight gain.^3^^,^^4^

The most frequent ocular complication of autologous hematopoietic stem cell transplantation is opportunistic infection of the ocular adnexa, cornea, vitreous, or retina, occurring during the period of extreme neutropenia before engraftment.^5^ Ischemic retinopathy has also been reported.^6^ We describe here a case of severe bilateral periorbital edema in a patient after hematopoietic stem cell transplant for neuroblastoma.

## 2 Case report

A three-year-old girl developed rapidly progressive subcutaneous swelling in the left anterior temporal area. Magnetic resonance imaging showed a 5.8 cm expansile heterogeneous gadolinium-enhancing mass centered along the left sphenoid wing. It invaded the left orbit, displacing the lateral rectus muscle and the optic nerve (Fig. 1A). There were multiple enlarged pre-auricular and submandibular lymph nodes. Whole body computed tomography identified a suspected primary tumor in the left adrenal gland (Fig. 1B) and numerous osseous metastases. A biopsy of the temporal mass revealed neuroblastoma with a high mitosis-karyorrhexis index and *N*-myc amplification. The child received immediate treatment with topotecan and cyclophosphamide, followed by cisplatin and etoposide, and then vincristine, doxorubicin, and cyclophosphamide. The left cranial mass resolved. The primary tumor was removed by radical adrenalectomy.

**Fig. 1A:**
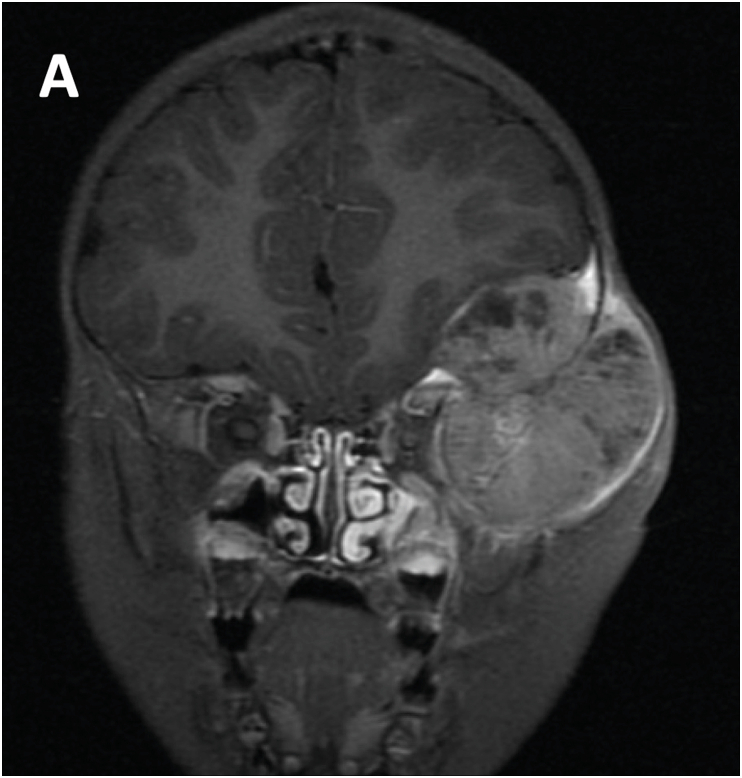
**Magnetic-resonance imaging of left anterior cranial mass.** T1 Flair coronal image obtained at the patient's initial presentation demonstrating an approximately spherical 5.5 cm diameter heterogenous mass centered on the left sphenoid wing. It exerts mass effect on the frontal lobe and invades the orbit, displacing the lateral rectus muscle and optic nerve medially.

**Fig. 1B:**
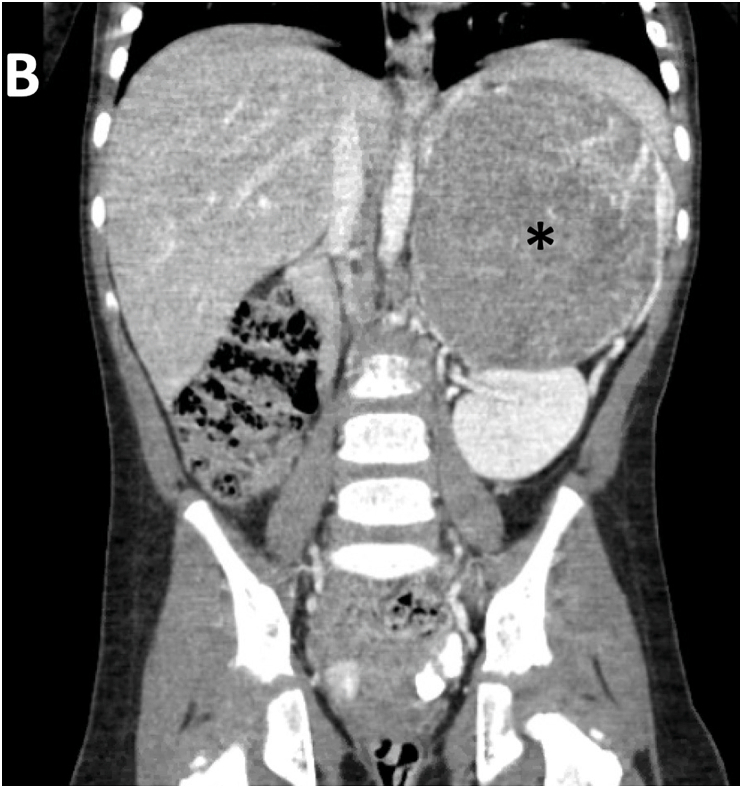
Computed tomography of abdomen. Contrast-enhanced imaging obtained at initial presentation identifying a large, well-circumscribed 9 cm left adrenal mass (*), just above the kidney.

The patient subsequently underwent tandem autologous peripheral blood stem cell transplants. Preceding the second transplant, myeloablative chemotherapy was intravenously delivered with melphalan, etoposide, and carboplatin. At the time of engraftment, nine days following stem cell transplantation, the patient developed fever, fluid retention, and weight gain from 14.7 kg to 16.3 kg, signifying the development of ES. The condition was treated with fluid restriction, chlorothiazide, furosemide, and hydrocortisone. Her extremity edema improved and her weight declined to 15.5 kg, allowing diuretic therapy to be reduced.

On post-engraftment day 11, 20 days after stem cell transplantation, the patient developed onset of bilateral periorbital edema. Her weight was 15.4 kg. The dosage of intravenous furosemide was increased. The next day, the Ophthalmology Service performed an evaluation. The child denied eye pain or any change in her vision. Chart review revealed that an episode of mild bilateral periorbital edema had occurred two months before, following the first autologous peripheral blood stem cell transplant. It improved in a few days without treatment.

On examination the patient had severe eyelid edema, more pronounced on the left side (Fig. 2A). The swelling involved the upper eyelids more than the lower eyelids. There was mild erythema, but no discharge or fluctuance. With effort the child could open the palpebral fissures a few millimeters. She was able to fixate and track a small target with each eye. The extraocular eye movements were full. Ocular alignment was orthotropic. In each eye, the conjunctiva was clear with no chemosis. The sclera was white and quiet. The cornea was lustrous and the anterior chamber was formed. The remainder of the exam was limited by lack of patient cooperation, and deferred per parental wishes.

**Fig. 2A:**
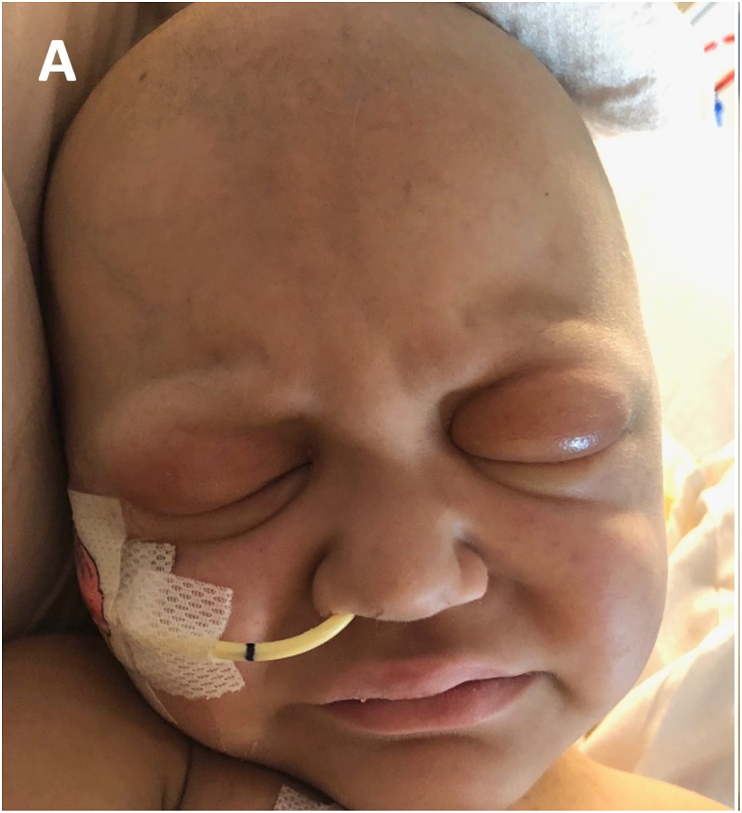
Bilateral periorbital edema. Marked edema of the upper eyelids, left worse than right, noted on initial ophthalmologic exam.

Orbital imaging was not obtained because of the requirement for sedation, and the history of a similar episode months earlier that resolved spontaneously, which suggested that the cause might be related to her hematopoietic stem cell transplant. A decision was made to monitor the patient closely with serial examinations, and to obtain imaging urgently if the eyelid edema persisted or worsened.

Two days following the development of periorbital edema, the patient was noted to have progressive hypertension, thrombocytopenia refractory to platelet transfusion, haptoglobin decrease, elevated lactate dehydrogenase, and proteinuria. Complement factor levels were normal, but liver function tests revealed elevated bilirubin levels. Chest radiography demonstrated pleural effusions and an echocardiogram showed mild dilation of the pulmonary arteries. Peripheral blood smear showed schistocytes. These findings fulfilled the diagnostic criteria for TA-TMA. Treatment with eculizumab, a monoclonal antibody that inhibits the complement cascade, was initiated. Ophthalmological exam three days later showed substantial improvement in the periorbital edema (Fig. 2B). However, the patient subsequently developed respiratory distress from bilateral pulmonary effusions and edema, cardinal manifestations of TA-TMA. Etanercept and rituximab were added to her treatment regimen. The patient remains hospitalized with active TA-TMA but no further ophthalmological manifestations.

**Fig. 2B:**
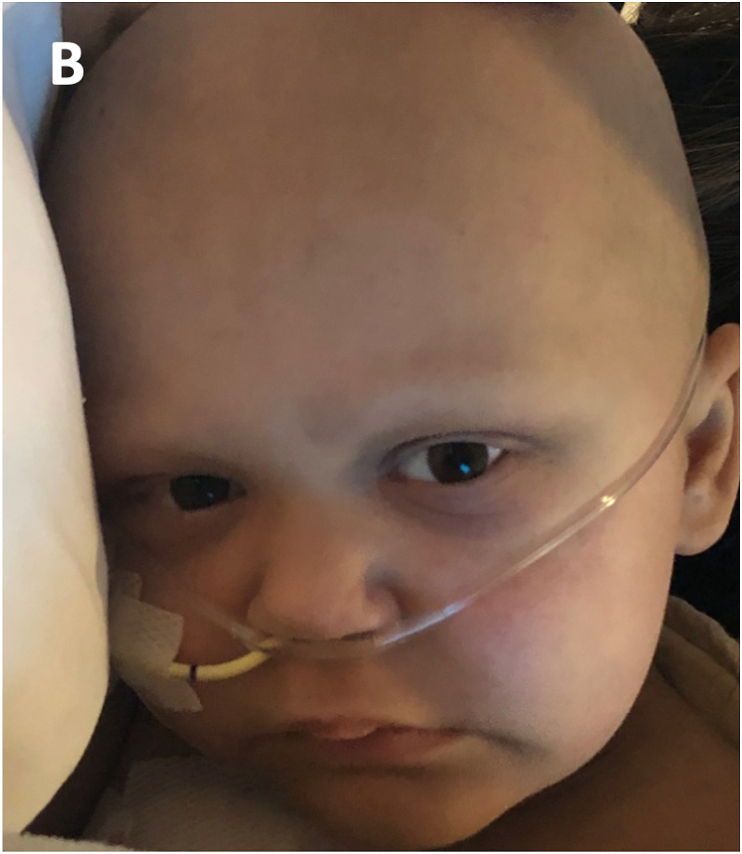
Improved periorbital edema. Resolved eyelid edema, three days after initial exam, after treatment for transplant-associated thrombotic microangiopathy had been initiated.

## 3 Discussion

We describe a child with metastatic neuroblastoma who experienced periorbital edema three weeks after hematopoietic stem cell transplant due to combined effects of ES and TA-TMA. A contributing role from chemotherapy is less likely but should also be considered.

Chemotherapy at myeloablative doses followed by hematopoietic stem cell transplantation has improved the prognosis for survival in neuroblastoma, one of the most common extracranial solid tumors of children.^7^ It is usually reserved for patients older than 18 months, with widespread metastases and amplification of the *N*-myc oncogene. These factors indicate a worse prognosis. It has been shown that two autologous stem cell transplants, delivered sequentially, result in a longer period of event-free survival than a single transplant.^8^

ES refers to a constellation of peri-engraftment complications that can follow hematopoietic stem cell transplantation. In this condition, cytokine release causes fever, rash, increased capillary permeability, weight gain, pulmonary edema, and elevated serum creatinine.^9^ These features were manifested in our patient and may account for the previous episode of mild periorbital edema that resolved with diuretics in her first post-transplant course.

ES has features that overlap with TA-TMA, another potentially life-threatening complication that can follow hematopoietic stem cell transplantation. The reported incidence of TA-TMA is between 7 and 39%, the wide range reflecting uncertainly regarding the diagnostic criteria.^10^, ^11^, ^12^, ^13^ Tandem stem cell transplants impart an even higher risk of developing TA-TMA.^10^ Diagnosis may be established through renal biopsy, which shows fibrin deposition, erythrocyte fragmentation, occluded microvasculature, and necrosis. The diagnosis is often made without tissue biopsy to avoid the risk of hemorrhage.^14^ Additional clinical manifestations indicative of TA-TMA include schistocytosis, elevated lactic dehydrogenase, decreased serum haptoglobin, proteinuria, hypertension, gastrointestinal bleeding, and polyserositis.^3^ To date, the only reported ophthalmological manifestation of TA-TMA is Purtscher-like retinopathy, presumably the result of thrombotic microangiopathy of the retinal vasculature.^15^^,^^16^

Volume overload is a less prominent feature of TA-TMA, although TA-TMA-associated renal dysfunction may contribute to alterations in fluid status; additionally, the endothelial dysfunction that underlies the pathogenesis of TA-TMA may increase vascular permeability, leading to third-spacing of intravascular fluid. Capillary leakage is thus a phenomenon that can occur in both ES and TA-TMA, with distinguishing factors being that ES presents earlier in the post-transplant course and TA-TMA has more prominent thrombotic features. Both of these conditions may have contributed to the development of periorbital edema in our patient.

The differential for periorbital edema is broad and includes infectious, inflammatory, neoplastic, and immunologic causes.^17^^,^^18^ Periorbital edema has also been described in thrombotic conditions similar to TA-TMA such as disseminated intravascular coagulopathy.^19^ Many of these processes converge on a pathway of leakage of fluid into the interstitial space, due to hypervolemia, hypoproteinemia, or increased vascular permeability. The thinness of eyelid skin and the relative laxity of the underlying subcutaneous tissues predispose to fluid accumulation in the periorbital area in these conditions. Chemotherapy, particularly imatinib, a tyrosine-kinase inhibitor, has also been implicated in the development of periorbital edema.^20^ Localized chemotherapy in the form of posterior sub-Tenon's injection of carboplatin is also associated with eyelid swelling.^21^ Our patient did not receive imatinib or periocular carboplatin.

Ophthalmologists are frequently called to evaluate post-transplant, immunocompromised patients in the inpatient setting. The index of suspicion for an infectious process is always high. In particular, acute onset of periorbital edema suggests a diagnosis of cellulitis or invasive fungal sinusitis. Another potential etiology is cavernous sinus thrombosis. Fever, which is usually present in ES, heightens the concern. This case highlights a noninfectious cause of eyelid edema in this population related to interstitial edema and identifies a previously unrecognized ophthalmic complication of hematopoietic stem cell transplant.

## Patient consent

The patient's parents consented in writing to publication of the case.

## Funding

This work was supported by grants EY029703 (J.C.H.) and EY02162 (Vision Core Grant) from the 10.13039/100000053National Eye Institute and by an unrestricted grant from 10.13039/100001818Research to Prevent Blindness.

## Authorship

All authors attest that they meet the current ICMJE Criteria for Authorship.

## Declaration of competing interest

The authors report no conflicts of interest.
